# Non-alcoholic Fatty Liver Disease as a Canonical Example of Metabolic Inflammatory-Based Liver Disease Showing a Sex-Specific Prevalence: Relevance of Estrogen Signaling

**DOI:** 10.3389/fendo.2020.572490

**Published:** 2020-09-18

**Authors:** Sara Della Torre

**Affiliations:** Department of Pharmaceutical Sciences, University of Milan, Milan, Italy

**Keywords:** NAFLD (non-alcoholic fatty liver disease), estrogens, estrogen receptors, liver, sex differences

## Abstract

There is extensive evidence supporting the interplay between metabolism and immune response, that have evolved in close relationship, sharing regulatory molecules and signaling systems, to support biological functions. Nowadays, the disruption of this interaction in the context of obesity and overnutrition underlies the increasing incidence of many inflammatory-based metabolic diseases, even in a sex-specific fashion. During evolution, the interplay between metabolism and reproduction has reached a degree of complexity particularly high in female mammals, likely to ensure reproduction only under favorable conditions. Several factors may account for differences in the incidence and progression of inflammatory-based metabolic diseases between females and males, thus contributing to age-related disease development and difference in life expectancy between the two sexes. Among these factors, estrogens, acting mainly through Estrogen Receptors (ERs), have been reported to regulate several metabolic pathways and inflammatory processes particularly in the liver, the metabolic organ showing the highest degree of sexual dimorphism. This review aims to investigate on the interaction between metabolism and inflammation in the liver, focusing on the relevance of estrogen signaling in counteracting the development and progression of non-alcoholic fatty liver disease (NAFLD), a canonical example of metabolic inflammatory-based liver disease showing a sex-specific prevalence. Understanding the role of estrogens/ERs in the regulation of hepatic metabolism and inflammation may provide the basis for the development of sex-specific therapeutic strategies for the management of such an inflammatory-based metabolic disease and its cardio-metabolic consequences.

## Introduction

### Liver Metabolism and Inflammation: Two Sides of the Same Coin

The liver is one of the most complex organs in the body, performing a multitude of functions, including the macronutrient metabolism, glucose, lipid and cholesterol homeostasis, protein and amino acid metabolism, detoxification and drug metabolism (1). The liver is also an immunological organ, being responsible for the production of acute phase proteins, complement components, cytokines and chemokines, and contains large, diverse populations of resident immune cells (2). Under physiological conditions, the liver is constantly exposed to dietary and gut-derived bacterial products with inflammatory potential and is engaged in tissue remodeling, all process requiring a tight regulation of the inflammatory response to maintain tissue and organ homeostasis and to redistribute the energy resources during the rising of an inflammatory response (3–5).

A close and coordinated regulation of metabolic and immune responses has been conserved through evolution, with lower and higher organisms sharing common ancestral structures and common key regulatory molecules and signaling systems (6). However, the integration between metabolic and inflammatory pathways have been set up in the context of nutrient limitations and have not evolved and adapted to the current habits and lifestyles, where overnutrition and the reduced physical activity lead to chronic disturbance of metabolic homeostasis and to aberrant immune responses (4, 7). The metabolic overload and the lack of metabolic homeostasis typical of obesity and obesity-associated metabolic diseases trigger a sustained and chronic inflammatory response, that, by converse, can disrupt systemic metabolic functions, thus fostering a vicious cycle that favors the progression of metabolic diseases (4). In the liver, the inability to resolve inflammation may lead to chronic pathological inflammation and to a disrupted tissue homeostasis, which can promote hepatic steatosis, fibrosis, cirrhosis, and liver failure (3, 5, 8–10).

Although the higher prevalence of obesity among female population, women result to be somewhat protected from the obesity-associated cardio-metabolic consequences, such as non-alcoholic fatty liver disease (NAFLD), at least until menopause (11). The reason of that likely relies on the tight regulation of metabolic and inflammatory processes that may have reached its maximum degree of complexity in the liver of female mammals, where the regulation of hepatic metabolism is under the control of sexual hormones, estrogens in particular, and is subjugated to the reproductive needs (12–15). In view of the tight link between energy homeostasis and reproduction, liver diseases show a sex-specific prevalence (16, 17) and are associated with reproductive dysfunctions in women (14, 18). Nowadays, changes in dietary and lifestyle habits as well as the increased lifespan of women, that spend more than 1/3 of their lives in post-menopause, can explain the increased incidence in female population of cardio-metabolic diseases, which are previously considered male-prevalent (16, 19, 20). In this view, research programs aimed to unravel the role of estrogen signaling in the regulation of metabolic and inflammatory processes may have a significant impact on the design of new therapies that can counteract the development of NAFLD and the associated cardio-metabolic consequences in a sex-specific fashion.

### NAFLD, a Canonical Example of Metabolic Inflammatory-Based Liver Disease Showing a Sex-Specific Prevalence

With respect to young, fertile women, men and post-menopausal women show an increased incidence of metabolic and inflammatory-based liver diseases (14, 18, 21). Among them, a canonical example is NAFLD, a syndrome characterized by excessive triglyceride (TG) accumulation within hepatocytes (22), that has reached epidemic proportions and represents an increasing public health issue due to its emerging association with several extra-hepatic diseases (23, 24), cardiovascular diseases (CVDs) in particular (25, 26). Indeed, cardiovascular mortality represents the commonest cause (45%) of death in NAFLD patients, followed by cancer (36%) and then liver-related mortality (7%) (27).

NAFLD is closely linked with peripheral insulin resistance and hepatic insulin resistance (28–30), a condition where insulin fails to suppress hepatic glucose production (HPG, which accounts for 90% of endogenous glucose production) but promotes lipid synthesis leading to hyperglycemia, hypertriglyceridemia and hepatic steatosis (31). Therefore, there is a significant correlation between HPG and the extent of liver fat in NAFLD patients (32) as well as between NAFLD and other metabolic insulin-resistant disorders such as type 2 diabetes mellitus (T2DM) (33, 34) and sarcopenia (35). Notably, women show an improved glycemic control, a greater peripheral and hepatic insulin sensitivity and a reduced HPG with respect to men (36–38), likely a consequence of a sex-dimorphic regulation of glucose homeostasis (39), to which the hepatic signaling of sexual hormones strongly contributes (40, 41), thus leading to a different susceptibility to NAFLD between the two sexes.

In the liver of NAFLD patients, TG accumulation it is due to increased *de novo* lipogenesis (DNL) (42, 43), increased delivery of fatty acids (FAs) to the liver (42, 44), and decreased lipid clearance consequent to impaired FA oxidation and lower lipid secretion (45, 46). Hepatocellular damage and fat-derived factors mediate the local activation of a pro-inflammatory response by hepatocytes and non-parenchymal cells, including Kupffer cells (KCs) and hepatic stellate cells (HSCs) (4, 47–49), that promote the recruitment of other immune cells, including neutrophils, T-lymphocytes and, mainly, macrophages (50).

The impaired mitochondrial oxidation (42, 51) and the up-regulation of both peroxisomal β-oxidation (52) and microsomal ω-oxidation (53) of FAs lead to chronic oxidative stress and result in the generation of reactive oxidative species (ROS) within the hepatocytes (42, 45). In addition to mitochondria—that are considered the most relevant source of ROS—and to peroxisomes and microsomes, the endoplasmic reticulum stress and enzymes as NADPH oxidase (NOX), cytochrome P450 2E1 (CYP2E1), cyclooxygenases, and lipoxygenases also produce ROS (54). According to the most valuable theories (22), the production of lipotoxic lipid intermediates and the excessive production of ROS further trigger a pro-inflammatory response that contributes to the progression of NAFLD to non-alcoholic steatohepatitis (NASH), fibrosis, cirrhosis and hepatocellular carcinoma (HCC) (46, 55, 56). Pro-inflammatory cytokines released by immune cells intensify the inflammatory process, that hinders the liver to orchestrate a proper tissue regeneration by replacing the hepatocytes subjected to cell death or apoptosis, as occurs under physiological conditions (57). Possibly as an unsuccessful effort against liver injury and tissue regeneration, HSCs become activated and differentiate into myofibroblasts, that, in turn, express actin and diverse types of collagen, leading to extracellular matrix deposition and fibrosis and liver degeneration (58–60).

In the presence of increased flux of free fatty acids (FFAs) and of chronic, low-grade inflammation, the liver acts as both a target of and a contributor to systemic chronic inflammation, triggering or boosting the progression of NAFLD and several extra-hepatic diseases (23), including atherosclerosis (61–63), cardiovascular diseases (62–64), chronic kidney disease (65), osteoporosis (66), and inflammatory bowel disease (67).

### From Metabolism to Liver Injury: Role of Obesity and Nutrients in NAFLD Development

Although several factors might contribute to hepatic steatosis, including genetic and epigenetic factors (68, 69), obesity represents the main trigger of NAFLD development and progression. However, independently of energy intake, also the macronutrient composition of the diet can be associated with NAFLD/NASH development (70). Different epidemiological studies have, therefore, demonstrated that dietary habits may directly promote NAFLD/NASH, by modulating hepatic TG accumulation and antioxidant activity and, indirectly, by affecting insulin sensitivity and the postprandial TG metabolism (70). Several studies have identified the overconsumption of fats (saturated fats and *trans*-fats, in particular) and sugars (fructose, in particular) as the main nutritional mediators of NAFLD development (71–73), while the role of proteins and amino acids in NAFLD etiology has been less investigated and still raises controversies (70).

#### Obesity

The rising trends in obesity has been linked with the increase in the incidence and severity of NAFLD, with an estimated global prevalence of 25–30% worldwide, rising up to 90% in morbidly obese patients (26, 74). In obese NAFLD patients, ~60% of hepatic FAs are derived from FFAs released by the adipose tissue as a consequence of an enhanced lipolysis and taken up by the liver *via* the increased uptake mediated by CD36 (cluster of differentiation 36) (75–77). To a less extent, hepatic lipid deposits derive from dietary FAs (~15%) and from increased synthesis of new lipids (~25%) from ingested carbohydrates that reach to a greater extent the liver due to the insulin resistance of the muscle (43, 75, 78). The exposure of hepatocytes to high lipid and carbohydrate levels promotes lipotoxicity and glucotoxicity, that, in turn, lead to mitochondrial defects, endoplasmic reticulum stress and oxidative stress (45, 79). The ectopic accumulation of lipid toxic intermediates triggers the activation of inflammatory pathways, cellular dysfunction, and lipoapoptosis, all features favoring NAFLD progression and liver injury (22, 80, 81).

Obesity also affects the liver through the unbalanced secretion of adipokines, exerting different effects on insulin resistance, hepatic steatosis, inflammation and fibrosis (82). For example, the obesity-associated reduction of adiponectin levels promotes insulin resistance and hepatic steatosis, while the increased levels of leptin foster hepatic inflammation (82). In the adipose tissue of obese people, the infiltration and activation of immune cells (macrophages, B-lymphocytes, T-lymphocytes and neutrophils) that produce pro-inflammatory cytokines (e.g., interleukin 1β, IL-1β; interleukin 6, IL-6; tumor necrosis factor-alpha, TNF-α) impair the dynamic antagonism between adipokines and cytokines and facilitate the progression of steatosis, inflammation and fibrosis (82).

Under obesogenic-like conditions, in addition to adipose tissue, the impaired regulation of metabolic process and signaling pathways in other tissues showing a strong interplay with the liver, including the skeletal muscle (83–85) and the gut-microbiota (86, 87), can further negatively affect the hepatic metabolic homeostasis and boost the progression of NAFLD.

In addition to genetic factors (88, 89), estrogen signaling strongly contributes to sex differences in obesity and associated cardio-metabolic consequences such as NAFLD (20, 21, 39, 90–93). With respect to pre-menopausal women, lean and obese men tend to accrue more visceral fat, that, having a greater lipolytic potential than subcutaneous adipose tissue, strongly contributes to increased FFA flux to the liver, where FFAs mediate insulin resistance and NAFLD pathogenesis (94, 95). After menopause, there is a redistribution of fat toward visceral depots and a lower inhibition of adipose lipolysis, all changes that fuel the FFA flux to the liver and increase the risk of developing NAFLD in post-menopausal women (92, 94).

Sex-specific and estrogen-mediated differences in obesity-induced NAFLD are ascribable also to impaired regulation of metabolic process in extrahepatic tissues showing a cross-talk with the liver, such as the adipose tissue and the skeletal muscle, that under obesogenic conditions display increased insulin resistance and increased inflammation that might further aggravate the hepatic dysmetabolism (96–106).

#### Dietary Sugars

Over the past century, the increased intake of added sugars, fructose in particular, is associated with increased incidence of hepatic steatosis and liver inflammation (107–109). Unlike glucose, ingested fructose by-passes the rate-limiting step of glycolysis and is preferentially metabolized by the liver, where it stimulates hepatic DNL acting mainly through SREBP1c (sterol regulatory element-binding protein 1c) and ChREBP (carbohydrate responsive element-binding protein), inhibits the mitochondrial β-oxidation of long-chain FAs, induces endoplasmic reticulum stress, and promotes TG formation and hepatic steatosis (73, 110, 111). Owing to the molecular instability of its five-membered furanose ring, fructose promotes protein fructosylation and formation of ROS, yielding to hepatocellular damage and to the development of a pro-inflammatory response (73).

Even after a single meal, fructose strongly up-regulates an inflammatory cascade through increased hepatic JNK (c-Jun N-terminal kinase) activity and induces hepatic insulin resistance, all effects occurring specifically in hepatocytes (112). Recent reports suggest that fructose can also induce liver inflammation by acting directly on inflammatory cells, where it drives the production of pro-inflammatory cytokines (IL-6 and IL-1β) that further promotes an aberrant lipid metabolism (107, 109, 112, 113). The high intake of fructose can also lead to gut microbiota dysbiosis and contribute to inflammation, insulin resistance and NAFLD progression (114).

The consequences of extended fructose consumption on liver health are different between the two sexes, with males being more responsive to fructose and showing higher hepatic postprandial DNL and higher prevalence of NAFLD compared to females (115–117). These differences can likely be a direct consequence of the sex-specific modulation of glucose metabolism (39, 118) and of the specific relevance of estrogen signaling in regulating hepatic glucose metabolism and in promoting insulin sensitivity (119–121), acting also through FGF21 (fibroblast growth factor 21) signaling (122, 123). Accordingly, high fructose intake exacerbates the progression of NAFLD in ovariectomized (OVX) female mice by enhancing liver cell destruction, macrophage accumulation, and progression of fibrosis, all negative effects that can be reverted by 17β-estradiol supplementation (121).

#### Dietary Fatty Acids

The increased intake of dietary FAs is strongly associated with obesity and the development of obesity-associated metabolic diseases, such as NAFLD (124–127). Dietary regimens enriched in fats contribute to increase the hepatic pool of FAs, where they promote DNL and the generation of lipotoxicity through a sustained oxidation (128). Dietary FAs influence NAFLD pathogenesis also by modulating the gene transcription of specific enzymes and regulating various metabolic pathways involved in lipid metabolism (129, 130). Modern western diets are particularly enriched in saturated and *trans* FAs that are particularly detrimental for hepatic health, because they induce insulin resistance and fatty liver and promote liver injury by altering the composition of plasma cell membrane, thus impairing cellular homeostasis and amplifying the already sustained inflammatory signaling, that, in turn, boosts insulin resistance and apoptosis (127, 128, 131, 132). Conversely, diets enriched in ω3 polyunsaturated FAs (ω3 PUFAs), such as the Mediterranean diet (133), may be particularly effective in counteracting the early stages of NAFLD (134), limiting insulin resistance, oxidative stress, DNL and TG deposition in the liver (135, 136) and preventing the development of liver-associated cardio-metabolic diseases (137). ω3 PUFAs exert anti-inflammatory actions by preventing the alteration of cell membrane phospholipid composition and the disruption of lipid rafts, by inhibiting the activation of NF-κB (nuclear factor-kappa B), by reducing expression of inflammatory genes and by activating PPARγ (peroxisome proliferator-activated receptor γ) (138).

With respect to the female counterparts, men and male rodents show a higher propensity of developing hepatic steatosis/NAFLD that derives from the combination of increased FA import, DNL, and storage of lipids within the liver and lower dietary FA oxidation and secretion (91, 139). By comparing control and LERKO (liver-specific Estrogen Receptor alpha KO) mice, a recent study demonstrates that the liver ability of females to cope with the excess of dietary lipids strongly relies on the activity of hepatic ERα, that confers to females a higher metabolic flexibility (91).

Different dietary fatty acid regimens can also change the composition and the *ratio* of FAs in liver plasma cell membrane in gender-specific manner, another mechanism that can further explain the sex-specific incidence of NAFLD (140).

Furthermore, maternal high-fat diet can promote and even program hepatic steatosis/NAFLD and liver inflammation of offspring in a sexually dimorphic manner by altering gut microbiota (141) that has been shown relevant for the achievement of hepatic sexual dimorphism (142).

#### Dietary Amino Acids

While the hazardous effects of high-carbohydrate and high-fat diets upon hepatic structure/function are well-recognized, the potential effects of dietary regimens enriched in proteins and amino acids (AAs) on hepatic health are partly clarified and still raise controversies. Indeed, while several studies show a beneficial role exerted by high-protein diets in reducing body weight and in reverting hepatic steatosis, other studies suggest that high-protein diets can instead promote the development of NAFLD (143). The reasons of these contradictory effects on liver health can be ascribable to differences in dietary regimens (e.g., diet composition and protein source) and on the functional status of the liver (143).

Among AAs, branched chain amino acids (BCAAs: leucine, isoleucine, and valine), that account for 20% of total protein intake (144), exert beneficial effects on hepatic health as they alleviate hepatic steatosis and liver injury and prevent hepatic fibrosis and the development of HCC in NASH mouse models (145, 146). By contrast, elevated circulating BCAAs are strongly associated with several metabolic disorders, including obesity and insulin-resistant metabolic diseases (147, 148). NAFLD patients show a low hepatic content of BCAAs, that changes with the progression of the pathology, likely as a consequence of impaired expression of hepatic BCAA-degrading enzymes (149, 150). Furthermore, a recent study demonstrates that plasma BCAA levels display sex-dimorphic changes with increasing severity of NAFLD, independently of BMI, insulin resistance and age (151), suggesting a sex-specific regulation of BCAA metabolism and/or a sex-specific role of BCAAs in NAFLD development, as supported by pre-clinical studies (91). Indeed, although their causative or associative role has not yet clarified, among AAs and several other metabolites, BCAAs result the pathway most affected in the liver of a mouse model of diet-induced obesity (91). Notably, the decrease in AAs and, especially, in BCAAs correlates with increased lipid deposition in the liver of male, but not female mice; in fact, when exposed to an excess of dietary lipids, female mice, contrary to males, preserve the hepatic AA homeostasis, an effect associated with the ability to counteract liver lipid deposition (91), suggesting that the metabolism of BCAAs might have a key role in driving hepatic steatosis in a sex-specific fashion. The female-specific ability to preserve BCAA homeostasis and counteract liver lipid deposition is dependent on hepatic ERα, as it is lost in LERKO female mice (91), and it is likely a consequence of an higher metabolic flexibility conferred by hepatic ERα, that, in the female liver, adapts the hepatic metabolism to hormonal status and to nutrient availability, amino acids in particular (13, 15, 152, 153).

### Sex Differences in NAFLD Onset, Development and Progression

NAFLD is more common in men, in whom it has a 2.0–3.5-fold higher prevalence than in fertile women; however, after menopause the incidence of NAFLD increases significantly to reach the levels seen in men, owing to the putative protective effect of estrogens (14, 17, 154). Indeed, gender-specific prevalence of NAFLD is related to age: while men commonly display an increasing prevalence of NAFLD during adulthood from young to middle-age, the prevalence of NAFLD in women occurs ~10 years later than in men, rising after the age of 50 years, peaking at 60–69 years, and declining after 70 years (16). This last trend indicates that the increased incidence of NAFLD in aging women relies more on the lack of estrogens than on aging *per se*, even though aging may exacerbate the progression of NAFLD by negatively impacting on metabolic (155) and inflammatory processes (156). According to this view, young oophorectomized women (157) as well as young women suffering of other reproductive dysfunctions characterized by altered estrogen levels (such as Polycystic ovary syndrome, PCOS) (14, 158) show increased incidence of NAFLD with respect to young fertile women.

Even if the exact etiology of NAFLD in post-menopausal women is still unclear, the association of NAFLD with the cessation of ovarian activity and with other ovarian dysfunctions such as PCOS (159) suggests that estrogens protect against its development and progression. Notably, with respect to their control counterparts, pre-menopausal, post-menopausal, and PCOS women with NAFLD exhibit a significantly lower concentration of serum 17β-estradiol, which is the principal active estrogen (158). Accordingly, hormone replacement therapy (HRT) reduces the risk of developing NAFLD for post-menopausal women (16, 160).

In pediatric populations, NAFLD prevalence is higher in boys than in girls (161), even though sex differences are less relevant with respect to adult population, suggesting that the achievement of complete sexual differentiation is required to accomplish the sex-specific prevalence and features of such a pathology. Such a hypothesis is sustained by several studies showing a strict association between puberty and features of NAFLD (162) and between earlier age at menarche and the prevalence of NAFLD later in life (163–165).

Although the prevalence of NAFLD is undoubtfully higher in men than women, less clear is the sex-specific incidence of liver injury associated with NAFLD progression to NASH and fibrosis. In fact, some studies suggest that women have a lower risk of developing NASH and fibrosis (166–169), while others do not find differences between the two sexes (170, 171) or, even, indicate that women are more susceptible than men to an inflammatory-driven degeneration of NAFLD toward more harmful conditions (172–176). Most of these studies, however, has several limitations and important potential bias, as they do not differentiate between pre- and post-menopausal women or do not often consider the timing/duration of menopause, which may give confounding and contradictory results (12). By converse, consistent with the hypothesis that estrogens exert beneficial effects on liver health, menopause, premature menopause and prolonged estrogen deficiency have been independently associated with significant fibrosis in women with NAFLD (177, 178).

NAFLD incidence is increased in obese people suffering of other obesity-associated cardio-metabolic diseases; nevertheless, several mechanistic and longitudinal studies have indicated that NAFLD is an independent risk factor for atherogenesis (179–181) and CVDs (23, 63, 182–184) apart from other metabolic disorders. Although still debated, the causal relationship independent of other metabolic risk factors seems to rely on the systemic inflammatory milieu initiated in part by liver-secreted cytokines and molecules (23, 63). In addition to enhanced inflammation, a growing body of evidence indicates that, along with NAFLD progression, the alteration of cholesterol and lipoprotein metabolism (185–187) and the excessive generation of ROS may lead to the accumulation of oxidized low-density lipoprotein (ox-LDL) in the liver (188–190) and to macrophages transformation into foam cells, which is a hallmark of atherosclerosis.

Given such a correlation between NAFLD and CVDs, it is not surprising that, while in the general population women are less prone to CVDs under the age of 50 years, after menopause, women lose this protection and show a higher risk of developing NAFLD and cardio-metabolic associated consequences (191–193).

### Sex Differences in the Regulation of Metabolism and Inflammation in the Liver

Sex-specific prevalence, progression and outcomes of hepatic diseases and their associated co-morbidities might be considered the resultant of sex differences typifying the male and female liver phenotype.

The liver is the major metabolic organ in mammals with the highest degree of sexual dimorphism (194, 195). Most of the sex differences in liver gene expression are dictated by the temporal pattern of circulating growth hormone (GH), which is sex dependent (highly pulsatile in males and more continuous in females) (196, 197) and under gonadal control (198–200). GH regulates the sexually dimorphic patterns of a large number of liver-expressed genes, including various plasma and urinary proteins, cytochromes P450 (CYPs, which contribute to sex differences in sex steroid hormone metabolism), enzymes devoted to steroid and foreign compound metabolism, and various receptors and signaling molecules involved in a broad range of physiological processes (194, 197, 201, 202). GH pattern carries out its sexual differentiating action of liver functions through multiple intracellular signaling pathways, including the transcription factor signal transducer and activator of transcription 5b (STAT5b) (203–205), hepatocyte nuclear factors 3β, 4α and 6 (HNF3β, HNF4α, HNF6) (206, 207) as well as their signaling cross-talk (208–210). GH dimorphic action on hepatic gene expression is also dependent on sex-specific regulation of DNA methylation and chromatin structure (197, 205, 211–215), resulting in major changes in sex-based liver functions. The hepatic responsiveness to GH dimorphic action changes during development (216–218) and remains dynamic during adult life (205, 217), charging the liver of the possibility to adapt its functions to the needs of the organism throughout life.

GH and its signaling pathway, acting mainly through insulin-like growth factor-I (IGF-I), regulate lipid metabolism in the liver (219) and play an important role in antagonizing NAFLD, by directly reducing DNL in the hepatocytes and by inactivating HSCs, therefore limiting fibrosis (220). According to this, GH deficiency in adults and in obese children is associated with increased prevalence of NAFLD and NASH, while GH replacement therapy improves these conditions (220, 221). In mice, the liver-specific ablation of the GH receptor (GHR) increases lipid uptake and DNL, resulting in hepatic steatosis that cannot be reverted by IGF-1 treatment (219).

GH and its signaling may have a key role also in the liver disease progression, by regulating excessive inflammation and allowing liver regeneration (222). By converse, during inflammation, the liver can become resistant to GH actions, through mechanisms involving proinflammatory cytokines such as IL-6, TNF-α, and IL-1β (223–226), thus worsening metabolic alterations.

In addition to the well-known dimorphic activity of GH, hepatic sexual dimorphism depends on several other factors, including genetic (213, 215) and epigenetic (227, 228) factors, diet (141, 229, 230), circadian rhythm (231, 232), gut microbiota (142) and sexual hormones (12, 152, 233).

In spite of the fact that our knowledge of the entity of hepatic sexual dimorphism under physio-pathological conditions remains very limited (12), several evidences, including the sex-specific prevalence, incidence, progression and outcomes of hepatic diseases such as NAFLD (17, 234, 235), indicate that, among the factors contributing to hepatic sexual dimorphism, estrogens and their receptors recover a key role.

Estrogens can regulate sex differences in the liver through direct and indirect mechanisms, that are both affected by and able to prevail over sex-based genetic background and sexual hormone-dependent regulatory activities. Estrogen activity can contribute to the sexual dimorphism of the liver directly (21, 91, 152, 236, 237) and indirectly, by regulating GH action, both in the central nervous system and locally (198, 233, 238–240). Several experimental models with impaired/lost estrogen signaling support the involvement of estrogen dependent pathways in the regulation of hepatic metabolism, also in a sexually dimorphic fashion (14, 91, 152, 153, 241).

Estrogen-mediated contribution of hepatic sexual dimorphism likely arises from different metabolic costs of reproduction and from higher metabolic flexibility acquired and perfected through evolution by the female liver of mammals to adapt the hepatic metabolism to nutrient availability to sustain the energy needs of reproductive function (12, 13, 152, 242). In view of these evidences, although androgens and androgen receptor (AR) contribute to the sex-based hepatic phenotype in a direct or indirect fashion, by acting on GH dependent pathways (200, 215, 233, 243) and by regulating the accessibility of DNA to several transcription factors through chromatin remodeling (244, 245), this review will focus in particular on the role of estrogen signaling in the regulation of metabolic-driven inflammatory process at the basis of NAFLD development and progression.

### NAFLD and Liver Inflammation

In NAFLD, the increased flux of FFAs, the generation of lipotoxicity and oxidative stress and insulin resistance concur in activating JNK and NF-κB signaling pathways, resulting in the increased production of pro-inflammatory cytokines, including IL-6 and TNF-α (4, 48, 49, 246). JNK is a member of mitogen activated protein kinases, which activation in fatty liver is associated with insulin resistance, activation of apoptosis and development of NASH (247–249). JNK pathway is differentially regulated between males and females during liver injury (250, 251), likely through an estrogen- and ERα-mediated inhibition of lipotoxicity-induced hepatic mitochondrial oxidative stress and, in turn, of JNK signaling pathway, thus avoiding the over-regulation of pro-inflammatory and pro-apoptotic process (252).

NF-κB is a transcription factor involved in innate and adaptive immune responses playing an essential role in the regulation of inflammatory signaling pathways in the liver. Under normal conditions, NF-κB is sequestered in the cytoplasm by the binding with IκB proteins; in response to stimulation by pathogenic stimuli, the degradation of the NF-κB inhibitor α (IκBα) allows the translocation of NF-κB to the nucleus, where it induces the expression of target genes encoding inflammatory mediators, such as TNF-α and IL-6 (4, 253). Persistent activation of the NF-κB pathway in the liver leads to a chronic inflammatory state and to insulin resistance, that further promote the development of NAFLD and NASH (81, 254). NF-κB and its downstream signaling pathway are under the inhibitory control of estrogen signaling (255–257), a regulation that accounts for sex- and menopause-associated over-regulation of hepatic inflammatory process and for the progression of NAFLD toward more harmful conditions such as NASH, fibrosis and HCC (16, 49, 230, 258, 259).

Homeostatic inflammation is tightly regulated by mechanisms acting to resolve inflammation in order to avoid excessive inflammation and pathological consequences. In the liver, the propagation or the resolution of inflammation mostly relies on the polarization abilities of KCs (the resident macrophages) and of the recruited macrophages (260, 261). Once activated by exogenous or endogenous danger signals, macrophages undergo pro-inflammatory or anti-inflammatory and reparative phenotype, respectively promoting or attenuating hepatic steatosis and liver injury in NAFLD (50, 258, 260, 261). As occurs in other physio-pathoplogical contexts (262, 263), estrogens might promote the skewing of pro-inflammatory macrophages toward anti-inflammatory macrophages and accelerate the resolution of inflammation and the tissue repair in the liver, thus contributing to limit NAFLD progression in pre-menopausal women with respect to men and post-menopausal women (258). Accordingly, a longer duration of estrogen deficiency increases the risk of developing fibrosis among post-menopausal women with NAFLD (177) as well as in OVX female mice fed with HFD (264).

Although the FA-induced activation of NOD-like receptor (NLR) NLRP3 inflammasome, which promotes IL-1β production, has been implicated in the progression of NAFLD to NASH (265–267), the potential role of estrogens in directly modulating NLRP3 inflammasome in the progression of NAFLD to NASH has been very poorly investigated (268) and remains unclear. By converse, estrogens suppress HCC through the ERβ-mediated upregulation of the NLRP3 inflammasome (269), likely contributing to the sex differences in HCC prevalence (270).

### Liver Regeneration and Inflammation

Inflammation triggers many chronic and degenerative diseases, but it also aims to eliminate damaged cells and initiate tissue repair and regeneration, through highly conserved mechanisms (271). Tissue repair and regeneration is particularly important for the liver, especially in response to injury, an ability essential for the maintenance of the hepatic metabolic functions (57).

The process of liver repair and regeneration relies on the proliferative capacity of existing mature hepatocytes in response to environmental cues and can be divided in two phases: a “priming phase,” where inflammatory mediators (e.g., IL-6, TNFα) trigger the inflammation-induced regeneration, and a “proliferation phase,” where mitogens (including hepatocyte growth factor, HGF; transforming growth factor-α, TGF-α; epidermal growth factor, EGF) and auxiliary mitogens (including bile acids; endothelial growth factor, VEGF; insulin-like growth factor system, IGF system; estrogens) carry out the proliferation of hepatocytes, also through the interaction with the liver-resident immune cells (272–275).

Among inflammatory mediators, IL-6 plays a key role in the liver regeneration, as it is responsible for activating ~40% of the genes that are immediately activated by transcription factors following partial hepatectomy (276, 277). According to that, mice lacking IL-6 show reduced hepatocyte proliferation, that can be restored with IL-6 administration (275). In addition to IL-6, also TNF-α is involved in the priming phase of liver regeneration, which requires the expression of inducible nitric oxide synthase (eNOS) to block the potential pro-apoptotic effect of TNFα signaling and trigger liver regeneration (5, 275). IL-6 and TNFα are released mainly by KCs, thus promoting hepatocyte proliferation. The KCs activation is mediated through the NF-κB signaling pathway triggered either by lipopolysaccharide (LPS)/Toll-like receptor4 (TLR4) signaling or by the components of the complement system like C3a and C5a (274, 275). While KC depletion is associated with impaired liver regeneration, the depletion of other liver-resident immune cells such as NK (natural killer) cells enhances liver regeneration due to reduced production of TNFα and IFNγ (interferon-γ), a negative feedback mechanism aimed at regulating the process of liver regeneration (5).

Males and females differ for their ability to regenerate the hepatic tissue in response to injury, with male animals showing a time-delay in the recovery process associated with a higher recruitment of monocytes (278), a difference that depends on both, estrogen and androgen signaling pathways (279–281). In regenerating livers, estrogens act mainly through ERα (281, 282), but also through ERβ (279), with ERα and ERβ orchestrating cell proliferation and differentiation, respectively. The relation between estrogens and IL-6 could be particularly complex, being IL-6 able to influence estrogen levels and, therefore, estrogen-dependent modulation of liver regeneration process (275).

A recent study demonstrated that estrogen and ERα might play an important role also in the accumulation of fats in the liver by modulating CD36 during the early phase of liver regeneration, when fatty acids, triglycerides and cholesterol are required for the proliferation of hepatocyte and for the formation of new cell membrane (283).

### The Lack of Estrogen Signaling Impairs the Regulation of Hepatic Metabolism and Inflammation: Lessons From Estrogen Deficient and Knockout Mice

#### Estrogen Deficiency in Females

The relevance of estrogen signaling in the regulation of female hepatic metabolism and inflammation has been investigated in several pre-clinical studies recapitulating the effects of estrogen deficiency observed in post-menopausal women (14, 18, 169, 284, 285).

In the liver of ovariectomized (OVX) female mice, the lack of estrogens leads to hepatic insulin resistance, to enhanced DNL and FA import, and to reduced FA oxidation and secretion, resulting in increased body weight and fat mass and in fatty liver (14, 21, 153, 286, 287). In OVX females, the administration of estrogens improves insulin sensitivity and suppresses gluconeogenesis *via* the transcription factor FOXO1 (Forkhead Box O1) (288), prevents hepatic fat deposition by inhibiting DNL (153, 289), facilitates the VLDL (very low density lipoprotein)-mediated export of lipids from the liver by increasing hepatic VLDL-TG production and expression of microsomal triglyceride transfer protein (21, 290, 291) and sustains the β-oxidation of FAs by inducing expression of PPAR-α (peroxisome proliferator-activated receptor α) and FGF21 (123, 289).

Although estrogen replacement has been shown effective in reducing hepatic steatosis (123, 153, 287, 289, 291), however, the administration of constant amount of estrogens or SERMs (selective estrogen receptor modulators) partially restores a proper regulation of hepatic metabolism (123, 292). The reason for that likely resides on the fact that the administration of constant amount of estrogens does not reproduce the physiological oscillation of estrogen levels typical of the reproductive cycle and, therefore, fails to reproduce the cyclic activation of hepatic ERα, which is responsible for a tuned modulation of hepatic metabolism in females (15, 153, 292).

Moreover, estrogens may have a significant impact on hepatic metabolism depending on their route of delivery. For example, while transdermal estradiol reduces plasma TGs by increasing the rate of VLDL-TG clearance without affecting VLDL-TG production (293, 294), oral delivery of estradiol increases VLDL production and plasma TGs, indicating the liver the most responsible of estrogen's effects on increasing VLDL-TGs (21).

The lack of estrogens is associated with increases in lipotoxicity, pro-inflammatory cytokines (e.g., TNFα, IL-1β, and IL-6) and oxidative stress and with decreases in anti-inflammatory cytokines (e.g., IL-10, interleukin 10) and antioxidant defense, all changes that can be reverted or, at least, mitigated by HRT (295–297). When exposed to high intake of dietary lipids, the liver of OVX female mice displays increased expression of *Mcp-1* (monocyte chemoattractant protein-1) and *Ccr2* (monocyte chemokine receptor 2) that trigger the recuitment of macrophages and promote hepatic fibrosis, endoplasmic reticulum stress and apoptosis, all changes that are improved by estradiol treatment (264).

#### Estrogen Deficiency in Males

Even in the liver of males, estrogen action is relevant for the regulation of glucose homeostasis, insulin sensitivity, lipid metabolism, and in the prevention of hepatic steatosis (298, 299). Estrogen deficiency in men with mutations in the gene codifying for aromatase (CYP19A1, the enzyme converting testosterone in estrogen) show impaired glucose and lipid liver metabolism (300, 301). Aromatase KO (ArKO) mice display increased adiposity, glucose intolerance and insulin resistance in both sexes (302); in male ArKO mice, increased insulin resistance is primarily due to increased hepatic gluconeogenesis through the induction of *G6Pase* (glucose 6-phosphatase) and *Pepck* (phosphoenolpyruvate carboxykinase) expression (299). By contrast, only ArKO males, but not females, show impaired lipid and lipoprotein metabolism and develop hepatic steatosis (302, 303). The administration of estrogens reverses the hepatic steatosis, by reducing the expression of genes involved in DNL (e.g., *Fasn*, fatty acid synthase; *Acaca*, acetyl-CoA carboxylase α; *Scd-1*, stearoyl-CoA desaturase-1) and fatty acid uptake (e.g., *Adrp*, adipocyte differentiated regulatory protein) (302, 304) and by restoring the expression of enzymes involved in FA oxidation (e.g., *Cat*, catalase; *Mcad*, medium-chain acyl-CoA dehydrogenase) (305). Although the precise mechanism of estrogen action in the liver of males have not been fully elucidated, studies performed in KO mice suggest that estradiol mediats PPARα signaling in protecting against hepatic steatosis (306, 307).

Estrogen deficiency in ArKO males is also responsible for increased hepatic mitochondrial apoptosis and altered permeability of the mitochondrial membranes, that are restored by supplementation of 17β-estradiol (308).

#### ERα in Females

Estrogens can mediate their biologic effects in the female liver acting mainly through the estrogen receptor α (ERα, the isoform most expressed at the hepatic level) (15, 152) through a number of mechanisms, including the regulation of gene transcription by the direct binding to specific estrogen responsive elements (ERE) or by the tethering with other DNA-binding factors and by non-genomic action through membrane-associated ERα (15, 153, 309–313).

While the lack of ERβ does not affect hepatic phenotype (314), the role of ERα in the regulation of hepatic metabolism and inflammation has been highlighted by several studies performed with total body (ERαKO) and liver-specific (LERKO) ERα knockout mice (311). ERαKO mice mostly recapitulate the metabolic phenotype of OVX animals, with increased body weight, visceral adiposity, glucose production, insulin resistance, and hepatic steatosis associated with increased hepatic inflammatory signaling (21, 314–316). Differently from control mice, ERαKO mice are not able to antagonize the induction of cytokines in consequence to a pro-inflammatory stimulus, indicating that ERα protects the liver against liver inflammation (317).

The LERKO mouse represents a useful tool to elucidate the specific relevance of ERα in the liver, especially in the hepatocytes, as this mouse model has been obtained by crossing mice expressing *floxed* ERα with mice expressing *Cre*-recombinase under the control of albumin promoter, that it is specifically expressed in the hepatocyte cells (13). Although improperly, LERKO can be considered as liver-specific ERα KO mice, being hepatocytes the most abundant cell type in the liver (57) and being ERα the receptor for estrogens most expressed in the hepatocytes (152, 241).

Compared to control counterparts, LERKO females show an impaired regulation of genes relevant in the regulation of hepatic lipid and lipoprotein metabolism during estrous cycle progression (15), with aging and after ovariectomy (153). As a consequence, LERKO females show increased deposition of lipids in the liver and an impaired regulation of lipoprotein synthesis, leading to a reduced cholesterol efflux to the liver, impaired hepatic cholesterol clearance, high circulating cholesterol levels and increased susceptibility to atherosclerosis (15, 237).

Additional studies have confirmed the role of ERα in preventing hepatic steatosis by showing that liver-specific knockdown of ERα is sufficient to induce hepatic steatosis through a mechanism that seems to involve the regulation of small heterodimer partner (SHP), a transcription factor important in the regulation of hepatic metabolic processes and in the protection against hepatic inflammation (318, 319).

The livers of LERKO mice exhibit a greater expression of genes involved in the inflammatory process (e.g., *Tnf*α; *Il-1*β; interleukin-12 beta, *Il-12*β*; Ccr2)* and collagen deposition (sequestosome1, *Sqstm1*; vimentin, *Vim*; serpine1, *Serpine*); according to that, LERKO females display portal infiltration of mononuclear leukocytes and portal or centrilobular collagen deposition in the liver (15).

The action of the hepatic ERα is particularly relevant when mice are subjected to excess of dietary lipids: with the lack of hepatic ERα, LERKO females result no more protected against the excess of dietary lipids and accumulate lipids in the liver (91), a condition resembling what happens in OVX mice and post-menopausal women (14). However, differently from control OVX females, estrogen treatment fails to prevent lipid deposition in the liver of LERKO females, further stressing the specific relevance of hepatic ERα in the regulation of female hepatic metabolism (287, 320).

Also transgenic mice in which the expression of ERα is limited to the cytoplasm develop hepatic steatosis (312), suggesting that the protective effects of estrogens on liver health can be mediated by both, classical and non-nuclear mechanisms (321, 322).

#### ERα in Males

Similar to females, ERαKO male mice develop insulin resistance, impaired glucose tolerance, increased adiposity and marked hepatic steatosis (314, 315, 323). Despite its reduced expression compared to females (15, 152), also in males the liver-specific disruption of ERα signaling leads to altered expression of genes involved in carbohydrate and lipid metabolism (241, 324–326). Hepatic ERα plays a key role in the maintenance of hepatic metabolism, by suppressing hepatic gluconeogenesis and by decreasing DNL through its direct binding to the promoters of genes involved in gluconeogenesis (e.g., *Pepck, G6Pase*) and lipid metabolism (e.g., *Fasn, Acaca)* (241) and through the modulation of FOXO1 phosphorylation (326). As a consequence of the lack of ERα-dependent regulatory activity, LERKO males display elevated hepatic glucose production (HGP), liver insulin resistance, increased hepatic lipogenesis and liver lipid deposition (241, 326).

Recent studies suggest that hepatic ERα is required for the estrogen-mediated programming of the hepatic metabolism of males, contributing to hepatic sexual dimorphism (152) and accounting for the sex-specific metabolic response to diets enriched in lipids (91). In the liver of males, ERα is required also for optimal immune-metabolic function, as its lack causes increased expression of several inflammatory genes (327).

#### GPER

In addition to membrane localized ERs, estrogens can signal through G-protein coupled Estrogen Receptor (GPER, also called Gpr30), a cell surface receptor which role in the regulation of liver metabolism has recently emerged (328–330). After the binding with estrogens, GPER activate multiple non-genomic pathways, as well as the transcriptional programs through the regulation of target genes (331–334) in diverse cell types and tissues, including the liver (335).

GPER has been functionally implicated in several physiological and pathological process (335) and, in particular, in the regulation of metabolism (328, 332, 336–338) and immune response (339–341). In the liver, GPER plays a role in modulating lipid metabolism, in lowering circulating lipid levels and in reducing inflammation (20), as confirmed by several pre-clinical and clinical studies. Individuals carrying a hypofunctional genetic variant of GPER show increased plasma LDL cholesterol; according to that, the activation of GPER induces the expression of the LDL receptor (LDLR) in HepG2 liver cells (342). A recent study demonstrates that GPER mediates the estrogen-dependent reduction of LDLR degradation by preventing the internalization of PCSK9 (proprotein convertase subtilisin/kexin type 9), thus resulting in a higher LDL uptake by liver cells and, consequently, to lower circulating LDL cholesterol (343).

In OVX female mice, the activation of GPER lowers the levels of circulating lipids, reduces the expression of lipogenic and pro-inflammatory genes, and increases the expression of genes involved in lipid oxidation in the liver (329). In a KO mouse model, the lack of GPER leads to increased lipid accumulation in the liver and decreased circulating HDL levels in females, but not males (344), highlighting a sex-specific role of GPER in the metabolic homeostasis (329).

GPER signaling is associated with the immune and anti-inflammatory response, as revealed by its role in counteracting a variety of pathological conditions, including diabetes and obesity (330, 338, 345), atherosclerosis (346, 347), asthma (348), neuroinflammation (349, 350), and cancer (97, 351). In the liver, the lack of GPER enhances immune cell infiltration, fibrosis, and the production of inflammatory factors, such as IL-6, IL-1β, and TNFα in a mouse model of HCC (351). The activation of GPER signaling is effective in reducing the expression of IL-6, but not the viability and proliferation of hepatoma cells, suggesting that GPER action against hepatic tumorigenesis occurs through the regulation of inflammatory response rather than the direct modulation of tumor growth and invasion (351).

Although these studies suggest a direct involvement of GPER in the regulation of metabolism and inflammation in the liver, especially in females, it cannot be excluded that the hepatic effects due to the lack of its signaling are the results of a more complex interaction among metabolic tissues. Indeed, mice lacking GPER show increased adiposity, decreased insulin sensitivity, defective glucose/lipid homeostasis, and inflammation (337, 338, 352), all features that might indirectly affect the hepatic metabolism, pointing to the need of liver-specific GPER models to clarify the specific role of GPER in the hepatic tissue.

### Estrogens and Key Cell Types in Liver Metabolism and Inflammation

The liver is composed of several cell types, each of them having unique functions in the regulation of metabolism and immune response and showing interactions with the other cell type, thus cooperating at multiple levels in the regulation of the hepatic function. The major cell types contributing to the main liver functions are hepatocyctes, Kupffer cells, hepatic stellate cells, liver sinusoidal endothelial cells, and cholangiocytes.

#### Hepatocyctes

Hepatocyctes represent the most abundant cell type in the liver (accounting for 80% of liver mass) and are involved in several functions, including lipid and carbohydrate metabolism (353), protein synthesis (354), detoxification and drug metabolism (355, 356), and the secretion of coagulation and complement factors (353, 354, 357). In the hepatocytes, estrogens, mainly acting through ERα, limit gluconeogenesis (241, 288) preventing increased HGP and insulin resistance (288), limit the uptake of FFAs, inhibit DNL (153) and promotes FA oxidation (289) and export (287), thus preventing lipid deposition in the liver and the generation of lipotoxicity and ROS (252) that trigger a pro-inflammatory response acting as the driver of NAFLD progression and liver degeneration (81, 247). Estrogen signaling facilitates the resolution of inflammation by inhibiting the production of pro-inflammatory cytokines (264), regulates apoptotic process (358, 359), and promotes liver cell regeneration (279–282), thus limiting or preventing liver injury. As recapitulated by studies performed in OVX and LERKO females, the lack of the regulatory activity of estrogens in the hepatocytes favors the development and progression of NAFLD and, likely, of the associated cardio-metabolic diseases (e.g., atherogenesis) (16).

#### Kupffer Cells (KCs)

Kupffer cells (KCs) represent one-third of the non-parenchymal cells in the liver and account for 80–90% of tissue macrophages present in the body, acting as immune sentinels (360). KCs are important members of the innate and adaptive immune systems, serving as a first line of defense against bacteria, microbial debris and endotoxins derived from the gastrointestinal tract. Once activated, KCs trigger an inflammatory response by producing a panel of pro-inflammatory cytokines, including TNF-α, IL-1β, and IFN-γ, and provide to the clearance of phagocytosable particles (361). Given their role in the regulation of inflammatory and innate responses, KCs are considered as potential targets for the treatment of liver diseases, including NAFLD (360, 362, 363). Male and female KCs are different from a morphological (364) and functional point of view (258), contributing to sex differences in liver inflammation and regeneration (280) and in the prevalence and progression of NAFLD (17, 258), ALD (alcoholic liver disease) (365) and HCC (259, 366). Estrogens result involved in the sensitization of KCs to toxic stimuli (367) and in driving the pro/anti-inflammatory polarization of KCs, that exerts a key role in the resolution or progression of inflammation, thus counteracting or promoting the development of liver diseases (50, 368, 369). The estrogen-dependent regulation of cytokine production by KCs is predominantly mediated *via* ERα (370, 371), resulting the isoform most expressed in these cell types (372).

### Hepatic Stellate Cells (HSCs)

Although comprising only 5% of the liver cells, *hepatic stellate cells (HSCs)* play a central role in liver metabolism, especially in retinol metabolism and lipid storage (373). In healthy liver, HSCs are quiescent and store 80% of total liver retinol, that is released depending on its extracellular status. In injured liver, HSCs become activated and transform into myofibroblasts; activated HSCs lose their retinols and produce a considerable amount of extracellular matrix, thus leading to liver fibrosis (59). Although sex differences in the morphological expression of male/female HSCs has not been observed (364), several studies have demonstrated that estrogen inhibits the activation of HSCs and reduces liver fibrosis (374, 375), suggesting that estrogen signaling might account for the sex-specific prevalence of hepatic fibrosis. Although the molecular mechanism has not been fully clarified, estrogens seem to act through ERβ (376) and GPER (377), given that ERα is not expressed in these cells (378).

#### Liver Sinusoidal Endothelial Cells (LSECs)

Liver sinusoidal endothelial cells (LSECs), which comprise ~50% of liver non-parenchymal cells, are highly specialized endothelial cells containing many small pores or fenestrations, which provide open channels that facilitate the transfer of substrates between the blood and the liver parenchyma and regulate lipoprotein traffic to and from the hepatocytes (379, 380). Their unique morphology gives to LSECs a high endocytic capacity, enabling them to act as effective scavengers and promote the clearance of lipids and macromolecules and small particulates from the blood. The impairment of their function is associated with the development of extra-hepatic pathologies, including atherosclerosis (380). *LSECs* exert a key role in the innate and adaptive immunity, promoting the presentation of antigens and favoring the removal and clearance of circulating antigens and viruses (381). In addition to their roles as pathogen recognition and antigen-presenting cells, LSECs also have a critical role in the recruitment of leukocytes into liver tissue, thus influencing the composition of hepatic immune population. The balance between tolerance and effector immune responses driven by LSECs might promote the resolution or the progression of the immune response, eventually leading to several chronic liver diseases, including NAFLD, cirrhosis, fibrosis, liver failure and HCC (381, 382).

In LSECs, estrogens, even by modulating the levels and the nuclear occupancy of ER (372), enhance the production of nitric oxide (NO) and regulate the hepatic sinusoidal microcirculation (383, 384), likely explaining the higher incidence of liver cirrhosis with portal hypertension in men and post-menopausal women than pre-menopausal women (385).

#### Cholangiocytes

Cholangiocytes are the epithelial cells lining the intrahepatic and extrahepatic bile ducts; these cells participate in bile production and secretion and, although to a less extent than hepatocytes, have a role in the liver development, regeneration and repair (386, 387). Cholangiocytes can be activated by a variety of insults, including infections, cholestasis, and xenobiotics (386), leading to increased proliferation and to pro-fibrotic and pro-inflammatory secreted factors (388), that can favor the development of cholangiopathies and cholangiocarcinoma (389–392).

Cholangiocytes are targets of estrogen action: by acting through both ERα and ERβ and by activating either genomic or non-genomic pathways, estrogens play a key role in the regulation of proliferative and secretory activities of cholangiocytes (393, 394). The lack of estrogens in OVX females decreases the expression of ERs (2.5-fold for ERα and 35-fold for ERβ), leading to reduced cholangiocyte proliferation and bile duct mass; conversely, the administration of 17β-estradiol during bile duct ligation in OVX rats induced a normalization of bile duct mass, cholangiocyte proliferation, and apoptosis (395). Also in males, estrogens exerts a major role in stimulating cholangiocyte proliferation by preventing the increase of cholangiocyte apoptosis and loss of cholangiocyte proliferation (396). Notably, the altered expression and/or activation of ERα and ERβ is often associated with a high risk of primary biliary diseases (397–399).

Impaired bile flow leads to cholestasis, a pathology characterized by elevated levels of bile acid in the liver and serum followed by hepatocyte and biliary injury, that shows an increased incidence in women receiving estrogen for contraception or hormone replacement therapy, or in susceptible women during pregnancy. Although the molecular mechanisms involved in cholestasis remain controversial, recent findings suggest that estrogens may influence its course by directly modulating the patho-physiology of cholangiocytes, which are the primary target of damage in this disease (393).

Although each cell type plays a specific role in the liver and expresses a unique gene (400) and proteomic profile (401), only the cooperation among different cell types enables the liver to achieve its functions (402), a consideration that should be taken into account when performing *in vitro* studies, in which the cross-talk among liver cell types is lost or partially reproduced (403, 404). In this view, although challenging, the recent advances in the generation of human liver organoids might represent a potential, more reliable tool for the *in vitro* analysis of liver-specific biological processes and for disease modeling and drug screening at near-physiological conditions (405).

## Conclusion

**Figure 1:**
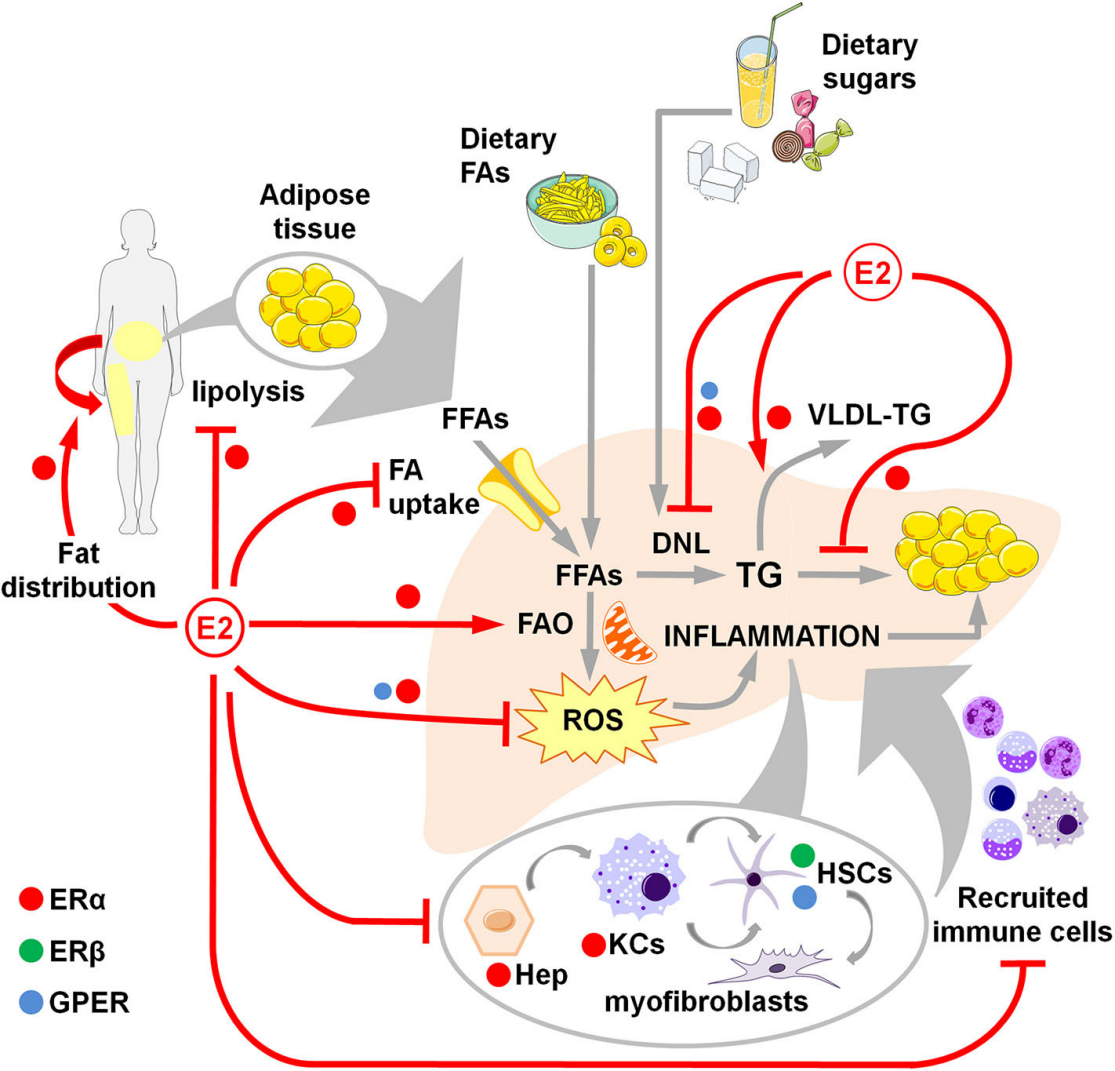
Overview of estrogen action through ERα, ERβ and GPER in counteracting NAFLD development and progression in women. Estrogens favor fat distributionr to subcutaneous deposits, inhibit adipose tissue lipolysis and reduce the uptake of FFAs, thus limiting the flux of FFAs to the liver. Estrogens limit dietary-induced DNL and facilitate the export of lipids as VLDL-TG. Estrogens promote the FA β-oxidation and prevent the activation of a sustained alternative FA oxidation that triggers lipotoxicity and the generation of ROS that, in turn, activate a pro-inflammatory response. Hepatocellular damage and fat-derived factors mediate the local activation of a pro-inflammatory response by hepatocytes, KCs and HSCs, that promote the degeneration of hepatic tissue and the recruitment of extra-hepatic immune cells that boost the inflammatory response and worsen the metabolic alterations. DNL, *de novo* lipogenesis; E2, estrogens (mainly 17β-estradiol); FAs, fatty acids; FFAs, free fatty acids; FAO, fatty acid oxidation; Hep, hepatocytes; HSCs, hepatic stellate cells; KCs, Kupffer cells; ROS, reactive oxygen species; TG, triglycerides; VLDL, very-low density lipoprotein.

The data summarized in this review outline the role of estrogens and their receptors in antagonizing the metabolic and inflammatory alterations that trigger and boost NAFLD development, thus determining its sex-dependent prevalence and its lower incidence in fertile females (Figure 1, Table 1).

**Table 1:** Summarizing the relevance of estrogen signaling, ERα, ERβ, and GPER in the sex-specific regulation of metabolic and inflammatory pathways relevant in NAFLD development and progression.

**Process/pathway**	**Regulation by estrogens**	**Mediators**			**Sex/Gender differences**	**References**
		**ERα**	**ERβ**	**GPER**		
Hepatic glucose metabolism	⦁	⦁			⦁	(39–41)
Hepatic glucose production (HPG)	⦁	⦁			⦁	(36–38)
Hepatic insulin sensitivity	⦁	⦁			⦁	(20, 120–123)
Hepatic FFA uptake	⦁				⦁	(91, 303)
Hepatic *de novo* lipogenesis	⦁	⦁			⦁	(91, 116, 303)
Hepatic FA oxidation	⦁			⦁	⦁	(91, 139, 303)
VLDL-TG export	⦁				⦁	(91)
Hepatic lipid storage and deposition	⦁	⦁		⦁	⦁	(91, 115–117, 303, 304, 329, 344)
Hepatic AA metabolism	⦁	⦁			⦁	(91, 151, 152)
Hepatic JNK activation	⦁	⦁			⦁	(251, 252)
Hepatic NF-κB activation	⦁	⦁			⦁	(255–257)
Macrophage polarization (from pro- to anti- inflammatory phenotype)	⦁	⦁	⦁		⦁	(258)
Liver regeneration	⦁	⦁	⦁		⦁	(278, 279, 281, 282)
Subcutaneous fat distribution	⦁	⦁			⦁	(92, 94, 95, 406)
Adipose tissue lipolysis	⦁	⦁			⦁	(92, 94, 406)

Estrogen-mediated effects likely arise from higher metabolic flexibility gained and perfected through evolution by the female liver to adapt the hepatic metabolism to the reproductive function (12, 13, 152, 242). Playing the liver the most relevant role in the accomplishment of energy requirements, during evolution the hepatic metabolism has been sharpened, in a sex-specific fashion, to reach an accurate interconnection of regulatory mechanisms aimed to sustain the energy needs of reproductive functions that are greatly different between the two sexes. The dynamic regulation of hepatic metabolism should have acquired a maximum degree of complexity in the liver of females that, compared to males, have to be more flexible in adapting their hepatic metabolism to the different, more variable, reproductive stages (reproductive cycle progression, pregnancy, lactation) that entail different energy requirements. The female liver had to develop and mold mechanisms able to sense and modulate efficiently the hepatic metabolism accordingly to the hormonal rhythm of estrogen fluctuations during the reproductive cycle and in other reproductive stages (pregnancy, lactation). In this view, in the liver of female mammals, estrogen signaling has therefore acquired a tight control on the hepatic metabolism through a sequence of well-tuned and intertwined events that have been perfectly tuned to secure reproduction only in favorable energy conditions and to support the energy needs of the different reproductive stages (14, 153, 235).

The high metabolic dynamicity conferred by estrogens to female liver contributes to prevent and limit the surge and progression of metabolic and inflammatory alterations in the liver, a mechanism underlying the increased incidence of NAFLD associated with the decline in liver metabolic flexibility after menopause.

The effects of estrogens and their receptors on the regulation of liver metabolism and inflammation may be direct or indirect, acting—for example—through other transcription factors and nuclear receptors (NRs) (407) with relevant and sex-specific activities in the liver (408) and in the NAFLD pathogenesis (409–411). Such an interplay might be particularly complex and regulated in the female liver: in view of its action in the regulation of reproductive process, ERα might have acquired in the female liver a regulatory role over these signaling pathways to adapt liver metabolism and inflammation to hormonal and nutritional status to accomplish the metabolic adaptations required to support the energy needs of reproduction. According with this idea, the lack of estrogens impairs the regulation of some NR signaling, including PPARα (289, 412) and glucocorticoid receptor (GR) (413), exerting pivotal roles in the regulation of hepatic metabolism and inflammation (414–416), thus favoring NAFLD development.

Despite the extensive, although probably underestimated, awareness on hepatic sex differences, the molecular mechanisms determining the sex-specific incidence of liver pathologies such as NAFLD are far to be unraveled. This knowledge has been prevented and affected by several limitations that stem from: (a) the paucity of available data on both sexes coming from pre- and clinical studies in which females are often underrepresented (12, 417); (b) the inability to enroll females in clinical studies (418–420); (c) the limited and, in some cases, misleading conclusions reached by experimental designs that did not take into account the relative contribution of genetic and hormonal backgrounds and exclude the sexual hormones as potential *confounding factors* (12, 417); (d) the lack of proper research tools helpful in investigating the genetic and/or hormonal factors relevant for the hepatic sexual dimorphism or the inability to use the available research tools in the best way (421, 422); (e) the fragmentary and still incomplete view coming from several studies that often do not share common protocols or lack of significance for the low number of the samples analyzed (422, 423); (f) the low, still insufficient commitment dedicated to dissemination of the results obtained from sex/gender research, such as educational programs addressed to health professionals (researchers, clinicians, scientific training programs, health institutions, etc.) and to society in general (419, 423–425); *g)* the still limited policies aimed at promoting sex/gender research programs (426–428). All these aspects contribute to our limited understanding of the nature and relevance of hepatic sexual dimorphism, thus preventing, so far, the development of more efficacious, sex-specific therapies against liver pathologies such as NAFLD, which incidence is greatly increasing in EU and accounts for €35 billions only in Germany, France, Italy, and United Kingdom (234, 429). Furthermore, the partial unawareness of the relevance of hepatic sexual dimorphism in the liver physio-pathology is a contributing cause to the development of associated cardio-metabolic diseases, such as atherogenesis and CVDs (430, 431). Similarly, the lack of sex-specific pharmacological treatments (that should differ in terms of molecules, dose, timing and risk of adverse drug reaction between the two sexes) leads often to drug-induced hepatotoxicity, representing the main cause of withdrawal of drugs from the market and the main reason of liver transplants (17).

In this view, a deeper understanding of the mechanisms underlining the sex-specific incidence of NAFLD and the role of estrogen signaling pathways will likely yield the basis for the design of more personalized hepatic therapies that would significantly improve the quality of life of a large section of our society as well as of men and women which experience impaired/lost hormonal signaling (i.e., due to gonadal failure, aging, exposure to endocrine disrupting chemicals).

## Author Contributions

SDT: figure preparation, writing, review, and editing.

## Conflict of Interest

The author declares that the research was conducted in the absence of any commercial or financial relationships that could be construed as a potential conflict of interest.
